# Supplementary material for: Brain Iron Deficiency Changes the Stoichiometry of Adenosine Receptor Subtypes in Cortico-Striatal Terminals: Implications for Restless Legs Syndrome
Source: Molecules. 2022 Feb 23;27(5):1489. doi: 10.3390/molecules27051489 (PMC8911604; doi:10.3390/molecules27051489)
Supplement: Supplementary file 1 [file molecules-27-01489-s001.zip › molecules-1601235-supplementary.pdf]

Supplementary Information

Brain iron deficiency changes the stoichiometry of adenosine receptor subtypes in cortico-striatal terminals. Implications for Restless Legs Syndrome

Rodrigues, Ferreira, Quiroz, Earley, García-Borreguero, Cunha, Ciruela, Köfalvi and Ferré

Table S1. Data and statistics for Figure 2

Animal #	A_1R CTRL	A_1R BID	$A_{2A}R$ CTRL	$A_{2A}R$ BID	$A_1R/A_{2A}R$ CTRL	$A_1R/A_{2A}R$ BID
1	58.5	66.4	4.42	15.0	13.2	4.42
2	85.8	67.2	6.63	8.49	13.0	7.92
3	48.7	52.2	3.14	15.4	15.5	3.39
4	61.0	53.6	2.57	5.66	23.7	9.48
5	31.2	64.3	1.79	20.9	17.4	3.08
6	47.6	22.4	18.2	5.10	2.62	4.40
7	68.3	36.0	17.2	5.24	3.97	6.88
8	83.2	40.7	15.2	6.48	5.46	6.28
9		36.7		6.46		5.69
10		66.9		20.5		3.26
11		39.1		14.7		2.67
**mean**	**60.54**	**49.60**	**8.65**	**11.26**	**11.85**	**5.22**
S.E.M.	*6.53*	*4.68*	*2.48*	*1.86*	*2.59*	*0.67*

Statistics for A_1R comparison

**Unpaired t test**	
P value	0.1787
P value summary	ns
Significantly different (P < 0.05)?	No
One- or two-tailed P value?	Two-tailed
t, df	t=1.403, df=17
**How big is the difference?**	
Mean of column A	60.54
Mean of column B	49.60
Difference between means (A - B) ± SEM	10.94 ± 7.802
95% confidence interval	-5.516 to 27.41
R squared (eta squared)	0.1037
**F test to compare variances**	
F, DFn, Dfd	1.416, 7, 10
P value	0.5962
P value summary	ns
Significantly different (P < 0.05)?	No
**Data analyzed**	
Sample size, column A	8
Sample size, column B	11

Statistics for $A_{2A}R$ comparison

**Unpaired t test**	
P value	0.4017
P value summary	ns
Significantly different (P < 0.05)?	No
One- or two-tailed P value?	Two-tailed
t, df	t=0.8601, df=17
**How big is the difference?**	
Mean of column D	8.651
Mean of column E	11.26
Difference between means (D - E) ± SEM	-2.607 ± 3.032
95% confidence interval	-9.004 to 3.789
R squared (eta squared)	0.04170
**F test to compare variances**	
F, DFn, Dfd	1.296, 7, 10
P value	0.6852
P value summary	ns
Significantly different (P < 0.05)?	No
**Data analyzed**	
Sample size, column D	8
Sample size, column E	11

Statistics for $A_1R/A_{2A}R$ comp.

**Unpaired t test with Welch's correction**	
P value	0.0385
P value summary	*
Significantly different (P < 0.05)?	Yes
One- or two-tailed P value?	Two-tailed
Welch-corrected t, df	t=2.478, df=7.937
**How big is the difference?**	
Mean of column G	11.85
Mean of column H	5.224
Difference between means (G - H) ± SEM	6.630 ± 2.676
95% confidence interval	0.4508 to 12.81
R squared (eta squared)	0.4361
**F test to compare variances**	
F, DFn, Dfd	10.94, 7, 10
P value	0.0011
P value summary	**
Significantly different (P < 0.05)?	Yes
**Data analyzed**	
Sample size, column G	8
Sample size, column H	11
